# Analysis of tumor vascularization in a mouse model of metastatic lung cancer

**DOI:** 10.1038/s41598-019-52144-2

**Published:** 2019-11-05

**Authors:** Ariunbuyan Sukhbaatar, Maya Sakamoto, Shiro Mori, Tetsuya Kodama

**Affiliations:** 1 Laboratory of Biomedical Engineering for Cancer, Graduate School of Biomedical Engineering, Tohoku University, 4-1 Seiryo, Aoba, Sendai, Miyagi 980-8575 Japan; 2 Biomedical Engineering Cancer Research Center, Graduate School of Biomedical Engineering, Tohoku University, 4-1 Seiryo, Aoba, Sendai, Miyagi 980-8575 Japan; 3 Department of Oral Diagnosis, Tohoku University Hospital, 1-1 Seiryo, Aoba, Sendai, Miyagi 980-8574 Japan; 4 Department of Oral and Maxillofacial Surgery, Tohoku University Hospital, 1-1 Seiryo, Aoba, Sendai, Miyagi 980-8574 Japan

**Keywords:** Cancer models, Metastasis

## Abstract

Therapies targeting tumor vasculature would improve the treatment of lung metastasis, although the early changes in vascular structure are incompletely understood. Here, we show that obstructive metastatic foci in lung arterioles decrease the pulmonary vascular network. To generate a mouse model of lung metastasis activation, luciferase-expressing tumor cells were inoculated into the subiliac lymph node (SiLN) of an MXH10/Mo-*lpr*/*lpr* mouse, and metastatic tumor cells in the lungs were activated by SiLN resection. Activation of metastases was monitored by *in vivo* bioluminescence imaging. Pulmonary blood vessel characteristics were analyzed using *ex vivo* micro-computed tomography. The enhanced permeability and retention (EPR) effect in neovasculature after tumor cell activation was evaluated from the accumulation of intravenously injected indocyanine green (ICG) liposomes. Metastatic foci in lung arterioles were investigated histologically. Micro-computed tomography revealed decreases in pulmonary blood vessel length, volume and number of branching nodes during the early stage of metastasis caused by metastatic foci. ICG liposome accumulation by the EPR effect was not detected. Histology identified metastatic foci in lung arterioles. The lack of an EPR effect after the formation of metastatic foci in lung arterioles makes conventional systemic chemotherapy ineffective for lung metastasis. Thus, alternative therapeutic methods of drug delivery are needed.

## Introduction

The prognosis for patients with cancer metastasis to the lungs is poor despite recent advances in therapy, such as the use of various drug combinations^1–3^. Indeed, the five-year survival rate for patients diagnosed with advanced-stage lung metastasis is only 5%. Metastatic foci in the lungs^4^ result from the hematogenous and/or lymphatogenous spread of a tumor originating in another part of the body, e.g., head and neck, colorectal or breast cancer^5–8^. The symptoms of lung metastasis depend on the locations and sizes of the tumor(s) in the lungs. The similarity of symptoms between lung metastasis and other pulmonary diseases often leads to late-stage diagnosis and delayed treatment, resulting in poor survival^9^.

The treatment of tumor metastases in the lungs is based on the cancer stage and symptoms, and available therapies include chemotherapy, surgery, radiotherapy or a combination of these^10^. However, a systemic assessment of the efficacy of chemotherapy, and in particular how efficacy is influenced by the presence of metastatic foci in pulmonary blood vessels, has not been reported. Systemically administered macromolecular drugs tend to accumulate in a tumor mass due to the increased permeability of blood vessels created by tumor-induced angiogenesis, which is referred to as the enhanced permeability and retention (EPR) effect^11^. EPR is considered to be the ‘gold standard’ method for tumor-targeting of systemically delivered drugs and is dependent on particle size, zeta potential, solubility and biocompatibility (routes of uptake and clearance)^12^. An important factor limiting research into the morphology of lung metastasis and the efficacy of treatment is the absence of a suitable animal model. Our research group has established a mouse model of lung metastasis using MXH10/Mo-*lpr*/*lpr* (MXH10/Mo/lpr) mice^13–15^, which involves the inoculation of tumor cells into a subiliac lymph node (SiLN) followed by resection of the tumor-containing SiLN at different time intervals^16–19^. Shao *et al*.^15^ proposed LN-mediated hematogenous metastasis after detecting lymphatic and venous system connections of SiLN by real-time observation of a fluorescent solution flow from the SiLN towards the efferent lymph node and thoracoepigastric vein; Takeda *et al*.^20^ observed these fluid dynamics in other strains of mice. Based on these results, that tumor cells metastasize from the primary tumor to the regional LNs, there is extracapsular spread into the veins located around the LNs. Although tumor cells may reach the secondary LNs through lymphatic vessels, it is the veins that are primarily responsible for the dissemination of these tumor cells throughout the cardiovascular system. Our research group observed the same phenomenon in other strains of mice. There are two main routes for tumor cells to flow out from the SiLN after injection of tumor cells. One is from the SiLN to the PALN via lymphatic vessels and the other is from the SiLN to the lungs via thoracoepigastric vein. Although there is a route from the PALN to the lungs via subclavian vein, we believe that the thoracoepigastric vein is dominant compared to the subclavian vein for the hematogenous route. Metastasis in the PALN progresses with time, depending on the tumor volume injected into the SiLN and the injection rate. Metastasis in the lung is activated by resection of lymph nodes. The process of activation of tumor cells in the lung may not be related to neoangiogenesis.

The aims of the present study were to evaluate whether metastatic foci in the lung impair the flow of systemically administered particles and/or molecules during the growth of pulmonary metastasis in a mouse model.

## Results

### Evaluation of pulmonary vascularization in the absence and presence of lung metastasis using micro-computed tomography (micro-CT)

**Figure 1:**
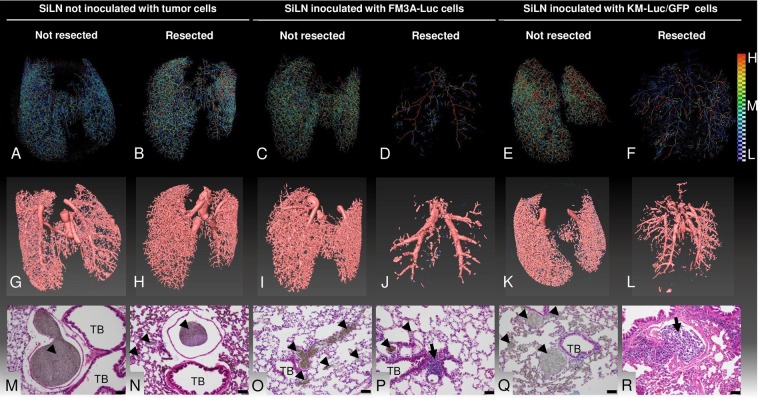
Reconstructed images of the pulmonary vasculature. (**A**–**F**) Skeletonized pseudocolor images of the lung vasculature. Lines representing each vessel centerline are shown using a color gradient from blue (low; **L**) to red (high, **H**). SiLN: subiliac lymph node. (**G**–**L**) Reconstructed images of the lung casts. (**M**–**R**) Lung sections stained with hematoxylin-eosin after micro-computed tomography scanning had confirmed metastasis in the blood vessels. Scale bar: 100 µm; arrow heads: contrast agent; arrow: tumor area. TB: terminal bronchioles.

**Figure 2:**
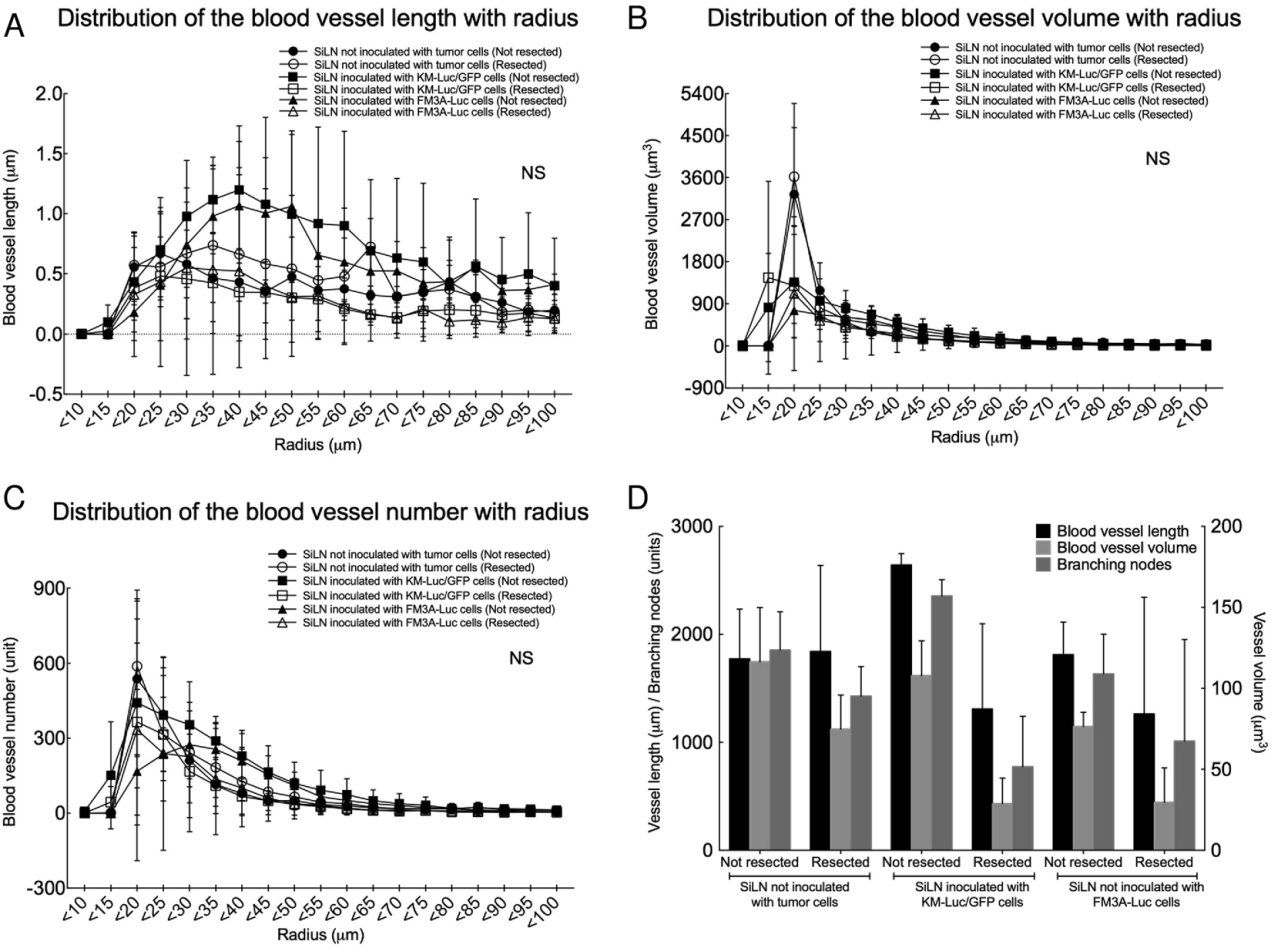
Characterization of lung blood vessel parameters. (**A**) Distribution of blood vessel length with radius. (**B**) Distribution of blood vessel volume with radius. (**C**) Distribution of blood vessel numbers with radius. (**D**) Effect of subiliac lymph node (SiLN) resection on lung blood vessel parameters in animals with a non-inoculated SiLN or with a SiLN inoculated with KM-Luc/GFP or FM3A-Luc cells. The left y-axis shows vessel length and the number of branching nodes, and the right y-axis shows vessel volume. SiLN not inoculated with tumor cells (Not resected): SiLN was not resected (*n* = 6); SiLN not inoculated with tumor cells (Resected): SiLN was resected (*n* = 6); SiLN inoculated with KM-Luc/GFP cells (Not resected): SiLN was not resected (*n* = 6); SiLN inoculated with KM-Luc/GFP cells (Resected): SiLN was resected on day 3 post-inoculation (*n* = 6); SiLN inoculated with FM3A-Luc cells (Not resected): SiLN was not resected (*n* = 6); SiLN inoculated with FM3A-Luc cells (Resected): SiLN was resected on day 3 post-inoculation (*n* = 6). All data are given as the mean ± SEM. There were no significant differences between any of the groups for all parameters.

First, we investigated pulmonary vascularization in the presence of lung metastasis using *ex vivo* micro-CT scanning (Fig. 1A–F, Videos S1–S6). For mice in which the SiLN was not resected (non-resection groups), the density of the lung skeleton was higher in animals with tumor cell-inoculated SiLNs than in control mice not inoculated with tumor cells (Fig. 1A,C,E). However, in mice in which the SiLN was resected (resection groups), the density of the skeleton was notably lower in mice that had received inoculation of tumor cells into the SiLN (Fig. 1D,F) than in non-inoculated mice (Fig. 1B). The reconstructed 3D casts for each experimental group are shown in Fig. 1G–L. Pulmonary blood vessel density was markedly lower in mice in which the SiLN was inoculated with tumor cells and then resected (Fig. 1J,L) but higher in mice in which the inoculated SiLN was not resected (Fig. 1I,K). Figure 1M–R show sections of lungs stained with hematoxylin-eosin (HE) after micro-CT scanning. The contrast agent was observed to fill the pulmonary blood vessels of control mice (both non-resection and resection groups) as well as mice for which the SiLN had been inoculated with tumor cells but not resected (Fig. 1M–Q). In contrast, mice in which the inoculated SiLN had been resected exhibited metastatic foci within the pulmonary blood vessels with no entry of contrast agent into the tumor masses (Fig. 1Q,R). Blood vessel length (Fig. 2A), volume (Fig. 2B) and number of branching nodes (Fig. 2C) were quantified from reconstructed 3D images. Analysis of the distribution of blood vessel length with radius (Fig. 2A) showed that the length of vessels with 30–75 µm radius was greater in the inoculated, non-resected SiLN group than in the inoculated, resected SiLN group. Moreover, the length of vessels with 30–75 µm radius was increased in the non-inoculated, resected SiLN group compared with the non-inoculated, non-resected SiLN group. Interestingly, in mice that received tumor cell inoculation into the SiLN, blood vessel length appeared to be longer in the non-resected SiLN group and shorter in the resected SiLN group. Observations of the distribution of blood vessel volume with radius (Fig. 2B) revealed that the blood vessel volume was higher in the non-inoculated, non-resected SiLN group than in the inoculated, non-resected SiLN group. In addition, blood vessel volume was lower in the inoculated, resected SiLN group than in the non-inoculated, resected SiLN group. Figure 2C shows the distribution of blood vessel numbers with radius. The number of blood vessels with a 30–55 µm radius seemed to be higher in the inoculated, non-resected SiLN group but lower in the inoculated, resected SiLN group.

Values for blood vessel length, blood vessel volume and the number of branching nodes within the entire lung are shown in Fig. 2D. Compared with the non-inoculated, non-resected SiLN group, the non-inoculated, resected SiLN group appeared to have a comparable blood vessel length but a lower blood vessel volume and a lower number of branching nodes. In mice that received inoculation of tumor cells, the blood vessel length, volume and number of branching nodes appeared to be smaller in mice with resected SiLNs than in mice with non-resected SiLNs. It should be noted that the perfusion and fixation methods did not induce any tissue shrinkage, and the 3D measurements were not affected by any triggers.

### Distribution of injected indocyanine green liposomes (ICG-LP) in mice with a solid tumor

Next, we used biofluorescence imaging to investigate the effects of ICG-LP size (Fig. S1C,D) on its time-dependent accumulation and loss based on the EPR effect (Fig. S2). A solid tumor was created by the injection of FM3A-Luc cells into the rear flank of the mouse. Figure S2Aa, Ac show luciferase activity in the solid tumor before the injection of ICG or ICG-LP, and Fig. S2Ab, Ad show the fluorescence intensity of ICG in the solid tumor after the intravenous injection of ICG or ICG-LP. A very high fluorescence intensity was detected 5 min after the injection of ICG-LP due to the accumulation of ICG-LP in the liver and solid tumor (Fig. S2Ad). Notably, fluorescence intensity was detected in the region of the solid tumor 48 h after the injection of ICG-LP, indicating that ICG-LP was retained in the solid tumor (Fig. S2Ad).

### Effect of SiLN resection on the distribution of injected ICG-LP in normal mice

Next, we evaluated whether resection of the SiLN had any effect on ICG-LP accumulation in the lung and other organs in normal mice, i.e., those in which the SiLN had not been inoculated with tumor cells (Fig. S2B–D). In normal mice that underwent SiLN resection, ICG-LP accumulation was observed in the region of the resected SiLN 6 h after ICG-LP injection (Fig. S2Cb). An *ex vivo* analysis of organs harvested 24 h after ICG-LP injection revealed no significant difference in ICG-LP accumulation in the lungs between the non-resection and resection groups (Fig. S2D), although a statistically significant difference between groups was observed for the spleen and proper axillary lymph node (PALN) (*P* < 0.001).

### Detection of metastasis and ICG-LP distribution in the lungs and PALN

The SiLN was resected on day 3 post-inoculation of tumor cells to induce metastasis in the lung. No luciferase activity was detected in the region of the SiLN after its removal.

**Figure 3:**
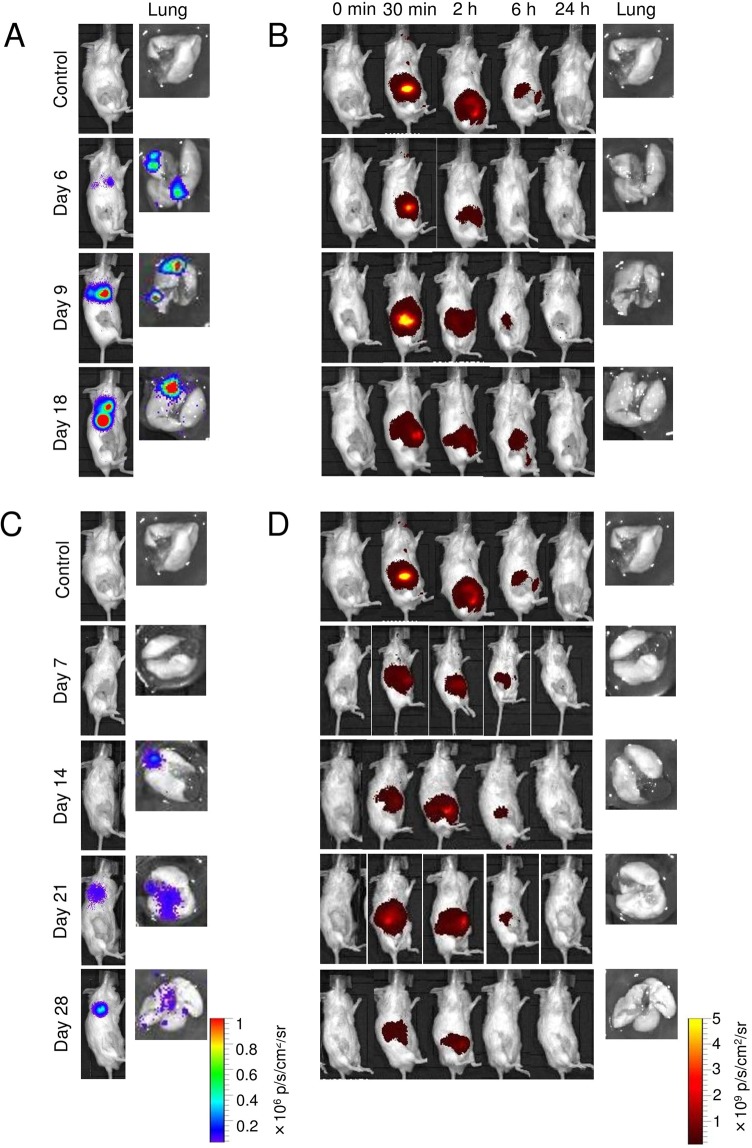
Evaluation of EPR effects in the metastatic lung. (**A**,**B**) Luciferase bioluminescence and indocyanine green (ICG) biofluorescence in experiments utilizing KM-Luc/GFP cells. (**C**,**D**) Luciferase bioluminescence and ICG biofluorescence in experiments utilizing FM3A-Luc cells. Tumor cells were inoculated into the unilateral subiliac lymph node (SiLN), and the SiLN was resected on day 3 post-inoculation, which activated tumor cells in the lung. ICG-LP was intravenously injected into the tail vein, and ICG-LP distribution was evaluated in the mouse *in vivo* and in the lungs *ex vivo* (the lungs were harvested 24 h after ICG-liposome [ICG-LP] injection). Bioluminescence images obtained *in vivo* were taken on the indicated days (**A**,**C**). Biofluorescence images obtained *in vivo* were taken before and 5 min, 2 h, 6 h and 24 h after ICG-LP injection (**B**,**D**). (**A**) Representative *in vivo* and *ex vivo* bioluminescence images of KM-Luc/GFP cells. Luciferase activity was measured on days 3, 6, 9 and 18 post-inoculation, and the representative *in vivo* images were taken before the ICG-LP injection in all groups. Luciferase activity was detected in the lungs on days 6, 9 and 18. (**B**) Representative *in vivo* and *ex vivo* biofluorescence images showing ICG-LP accumulation in mice inoculated with KM-Luc/GFP cells. Maximum ICG-LP accumulation was detected 30 min after ICG-LP injection. No ICG-LP fluorescence was detected in the lungs 24 h after ICG-LP injection. (**C**) Representative *in vivo* and *ex vivo* bioluminescence images of FM3A-Luc cells. Luciferase activity was measured on days 3, 7, 14, 21 and 28 post-inoculation, and the representative *in vivo* images were taken before the ICG-LP injection in all groups. Luciferase activity was detected in the lungs on days 14, 21 and 28. (**D**) Representative *in vivo* and *ex vivo* biofluorescence images showing ICG-LP accumulation in mice inoculated with FM3A-Luc cells. Maximum ICG-LP accumulation was detected 30 min after ICG-LP injection. No ICG-LP fluorescence was detected in the lungs 24 h after ICG-LP injection.

Metastasis in the lungs and PALN was measured using an *in vivo* bioluminescence imaging system (IVIS) at 6 h and on days 3 (before resection), 6 and 9 post-inoculation with KM-Luc/GFP cells (*n* = 51), and days 3 (before resection), 7 (after resection), 14, 21 and 28 post-inoculation with FM3A-Luc cells (*n* = 72), and day 6 for the control group. Luciferase activity in the thoracic region was observed on day 6 (Fig. 3A) and day 21 (Fig. 3C), respectively, for mice that had received inoculation of KM-Luc/GFP and FM3A-Luc cells into the SiLN. Metastasis in the lung was detected by *ex vivo* bioluminescence imaging on days 6, 9 and 18 for animals inoculated with KM-Luc/GFP cells and days 14, 21 and 28 for those inoculated with FM3A-Luc cells (Fig. 3A,C). Luciferase activity in the PALN (i.e., metastasis) was observed on days 9 and 18 for KM-Luc/GFP cells and days 7, 21 and 28 for FM3A-Luc cells (Fig. S5A,B). Mice injected with ICG-LP were monitored for 24 h with biofluorescence imaging to evaluate ICG-LP accumulation *in vivo*. No ICG fluorescence was detected in any of the groups 24 h after injection (Fig. 3B,D). Furthermore, for both tumor cell types, no ICG fluorescence was detected in the metastatic lung or PALN in the *ex vivo* experiments (Fig. 3B,D).

**Figure 4:**
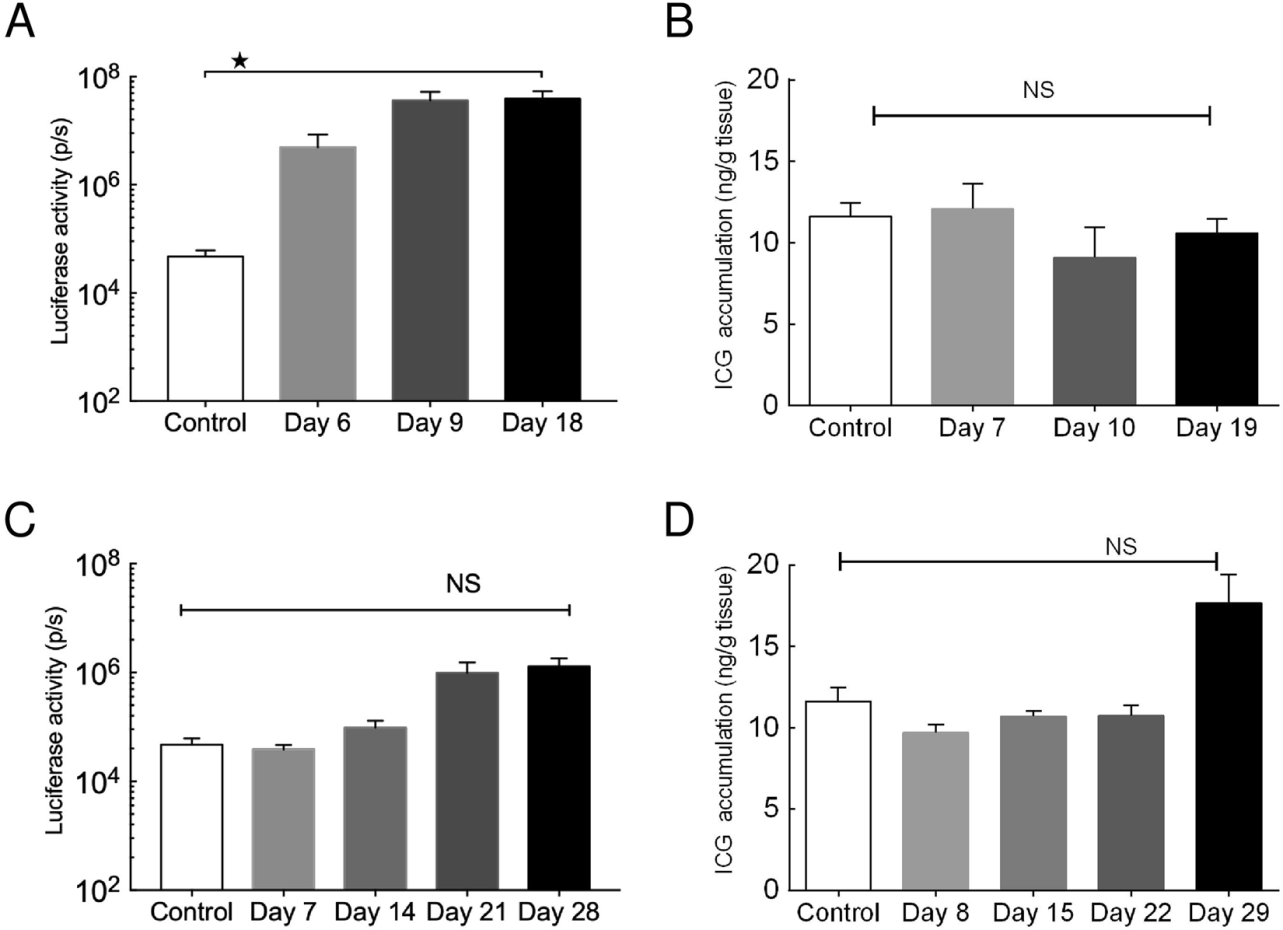
Quantitative *ex vivo* measurements of luciferase activity and indocyanine green (ICG) accumulation in the lung. Lungs were harvested 24 h after ICG-liposome (ICG-LP) injection, and ICG accumulation was quantified from the *ex vivo* lung fluorescence intensity as ng of ICG per g of tissue. (**A**) Luciferase activity in the lungs was measured *ex vivo* on days 6, 9 and 18 post-inoculation of KM-Luc/GFP cells (day 6, *n* = 8; day 9, *n* = 9; day 18, *n* = 5) and on day 6 for the control group (*n* = 4). The highest luciferase activity in the lungs was detected on day 18. Kruskal-Wallis test: *P < 0.05, control *vs* day 18. Data are given as the mean ± SEM. (**B**) ICG accumulation in the lungs of mice inoculated with KM-Luc/GFP cells. Lungs were harvested 24 h after ICG-LP injection, i.e., on days 7, 10 and 19 for the KM-Luc/GFP group (day 6, *n* = 8; day 9, *n* = 7; day 18, *n* = 9) and o*n* day 7 for the control group (*n* = 4). No significant differences were observed between groups. Data are given as the mean ± SEM. (**C**) Luciferase activity in the lungs measured *ex vivo* on days 7, 14, 21 and 28 post-inoculation of FM3A-Luc cells (day 7, *n* = 11; day 14, *n* = 10; day 21, *n* = 8; day 28, *n* = 6) and o*n* day 6 for the control group (*n* = 4). Numerically, the highest luciferase activity in the lungs was detected on day 28, although there were no significant differences between groups. Data are given as the mean ± SEM. (**D**) ICG accumulation in the lungs of mice inoculated with FM3A-Luc cells. Lungs were harvested 24 h after ICG-LP injection, i.e., on days 8, 15, 22 and 29 for the FM3A-Luc group (day 7, *n* = 6; day 14, *n* = 5; day 21, *n* = 6; day 28, *n* = 3) and o*n* day 7 for the control group (*n* = 4). No significant differences were observed between groups. Data are given as the mean ± SEM.

Luciferase activity in the PALN and lungs appeared to be time-dependent for both tumor cell types (Figs 4 and S3). Numerically, the highest luciferase activity in the metastatic lung and PALN was detected on day 18 for KM-Luc/GFP cells (Figs 4A and S3A) and day 28 for FM3A-Luc cells (Figs 4C and S3C). Furthermore, for KM-Luc/GFP cells, the luciferase activity on day 18 was significantly higher than controls for both the lungs and PALN (*P* < 0.05). For both tumor cell types, ICG accumulation in the lungs and PALN showed no significant difference over time (Figs 4B,D and S3B,D), although there appeared to be a trend towards an increase in ICG fluorescence on day 29 for FM3A-Luc cells (Figs 4D and S3D).

### Histopathological analyses

**Figure 5:**
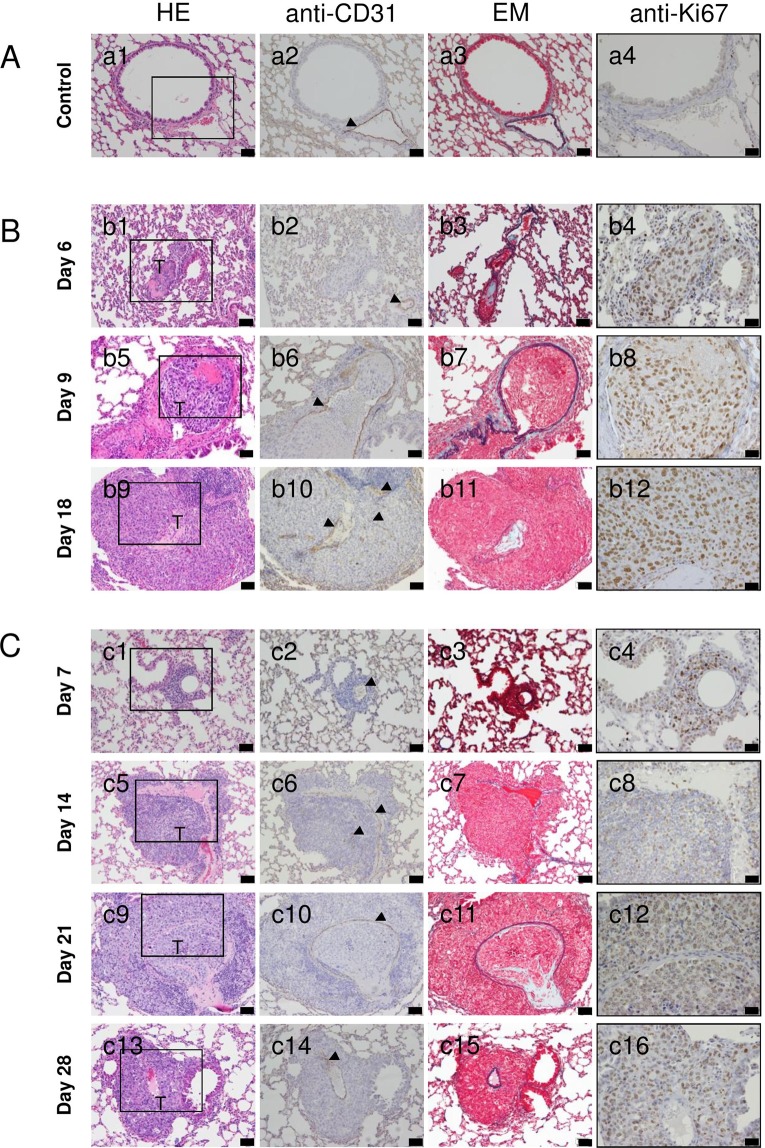
Histological analysis of the lungs. (**A**) Histological sections of the lung in control experiments. (**B**) Histological sections of the lung in experiments utilizing KM-Luc/GFP cells. (**C**) Histological sections of the lung in experiments utilizing FM3A-Luc cells. Tumor cells were inoculated into the unilateral SiLN, and the SiLN was resected on day 3 post-inoculation to activate tumor cells in the lung. Lungs were harvested on day 6 for control mice, on days 6, 9 and 18 post-inoculation of KM-Luc/GFP cells and on days 7, 14, 21 and 28 post-inoculation of FM3A-Luc cells. Metastatic foci were found in the blood vessels on days 6, 9 and 18 for KM-Luc/GFP cells and days 7, 14, 21 and 28 for FM3A-Luc cells. a1, b1, b5, b9, c1, c5, c9 and c13: hematoxylin-eosin (HE); a2, b2, b6, b10, c2, c6, c10 and c14: immunostaining of CD31; a3, b3, b7, b11, c3, c7, c11 and c15: elastic tissue-Masson’s trichrome (EM); a4, b4, b8, b12, c4, c8, c12 and c16: immunostaining of Ki67. Scale bar: 50 µm; T, tumor area; arrowhead, endothelial-positive cell. Boxes in HE-stained images (a1, b1, b5, b9, c1, c5, c9 and c13) outline the regions shown for Ki67 staining (a4, b4, b8, b12, c4, c8, c12 and c16).

**Figure 6:**
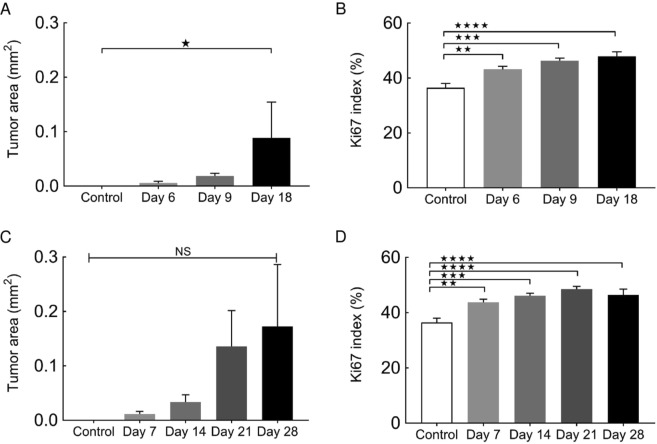
Quantification of histological analyses. (**A**) Tumor area in the lung for the KM-Luc/GFP groups was quantified from hematoxylin-eosin (HE) staining. Control (*n* = 4), day 6 (*n* = 4), day 9 (*n* = 12), day 18 (*n* = 10). Data are given as the mean ± SEM. Kruskal-Wallis test: *P < 0.05, control *vs* day 18. (**B**) Ki67 index of the lung for the KM-Luc/GFP groups was quantified from slides immunostained for Ki67 (*n* = 8 per group). Data are given as the mean ± SEM. Kruskal-Wallis test: **P < 0.01, control *vs* day 6; ***P < 0.001, control *vs* day 9; ****P < 0.000, control *vs* day 18. (**C**) Tumor area in the lung for the FM3A-Luc groups was quantified from HE staining. Control (*n* = 4), day 7 (*n* = 6), day 14 (*n* = 6), day 21 (*n* = 16), day 28 (*n* = 5). Data are given as the mean ± SEM. Tumor area in the lung appeared to increase with time, but there were no significant differences between groups. (**D**) Ki67 index of the lung for the FM3A-Luc groups was quantified from slides immunostained for Ki67 (*n* = 8 per group). Data are given as the mea*n* ± SEM. Kruskal-Wallis test: **P < 0.01, control *vs* day 7; ***P < 0.001, control *vs* day 14; ****P < 0.0001, control *vs* day 21 and control *vs* day 28.

Figure 5 shows representative lung sections obtained from animals with SiLNs inoculated with KM-Luc/GFP or FM3A-Luc cells. The sections were stained with HE (Fig. 5a1,b1,b5,b9,c1,c5,c9,c13) or Elastica-Masson (EM; Fig. 5a3,b3,b7,b11,c3,c7,c11,c15) or immunostained for Ki67 (Fig. 5a4,b4,b8,b12,c4,c8,c12,c16) or CD31 (Fig. 5a2,b2,b6,b10,c2,c6,c10,c14). Tumor cells were found in the pulmonary arterioles on days 6, 9 and 18 for KM-Luc/GFP cells (Fig. 5B) and days 14, 21 and 28 for FM3A-Luc cells (Fig. 5C). The boxes in the HE-stained sections highlight the regions shown for Ki67 immunostaining (Fig. 5a4,b4,b8,b12,c4,c8,c12,c16). No tumor foci were found in the lungs in the control group (Fig. 5A). The Ki67 staining percentage looks similar in all tumors (Fig. 5b4,b8,b12,c12,c16). The tumor area appeared to increase with time (Fig. 6A,C). The smallest tumor area in the lungs was detected on day 6 for KM-Luc/GFP cells (Fig. 6A) and day 7 for FM3A-Luc cells (Fig. 6C). Significant differences were not observed between groups for animals inoculated with FM3A-Luc cells, but a significant difference was found for control *vs* day 18 for animals inoculated with KM-Luc/GFP cells (*P* < 0.05). The Ki67 indexes of the lungs from mice inoculated with KM-Luc/GFP and FM3A-Luc cells are shown in Fig. 6B,D. Tumor cell proliferation was numerically highest on day 18 for KM-Luc/GFP cells and day 21 for FM3A-Luc cells. Statistically significant differences were found for control *vs* day 6, control *vs* day 9 and control *vs* day 18 for KM-Luc/GFP cells, and for control *vs* day 7, control *vs* day 14, control *vs* day 21 and control *vs* day 28 for FM3A-Luc cells (*P* < 0.01), respectively. Sections of the PALN stained with HE or immunostained for CD31 are shown in Fig. S4. Metastasis was detected in the PALN on days 9 and 18 for KM-Luc/GFP cells and days 7, 14, 21 and 28 for FM3A-Luc cells. It should be noted that significant weight loss did not occur during these experiments (Fig. S5).

## Discussion

Lung metastasis remains a serious public health challenge as a result of its frequency of occurrence, its lethality and the paucity of convenient models for exploring its pathological processes and potential therapeutic vulnerabilities. Despite recent advances, the fundamental features of lung metastasis, and especially its obstructive metastatic foci, are not fully understood. Here, we described the pathomorphology of lung metastasis in a mouse model in which tumor cells in the lung were activated by the resection of a tumor-bearing SiLN.

Micro-CT analysis indicated that tumor cells became trapped in an inactive state within lung blood vessels following their inoculation into the SiLN, but these cells became activated and continued to grow after resection of the tumor-bearing SiLN. The histopathology results revealed that metastatic foci were present in the pulmonary arterioles after SiLN resection (Fig. 1M–R), in agreement with this hypothesis. The pulmonary arterioles of normal mice, and mice in which the inoculated SiLN was not resected, were fully filled with contrast agent, whereas contrast agent was unable to flow through the lung blood vessels of mice with inoculated and (subsequently) resected SiLNs due to blockage of the vessels by tumor cells. These results also explain the pulmonary blood vessel defects observed in the metastatic lung (after resection of an inoculated SiLN) in the micro-CT experiments (Fig. 1G–L). In mice with a non-inoculated SiLN, blood vessel volume and number of branching nodes were smaller in animals with a resected SiLN than in those with a non-resected SiLN (Fig. 2A–C), implying that surgical resection of the SiLN affects blood vessel structure in other organs. Blood vessel length and number of branching nodes were greater in mice in which the SiLN was inoculated with tumor cells and not resected, compared with animals in which the SiLN was not inoculated or resected. However, in mice in which the SiLN was inoculated with tumor cells, blood vessel length, volume and the number of branching nodes were lower after resection of the SiLN compared with non-resection (Fig. 2A–C). It is likely that the total blood vessel length, volume and number of branching nodes were decreased in mice with inoculated and resected SiLNs due to vessel blockage by tumor cells and insufficient filling with contrast agent (Fig. 2D). The micro-CT imaging analysis revealed the simultaneous activation of multiple metastatic lesions in the lungs, and it became clear that tumor emboli had formed in the pulmonary arterioles and disrupted the blood circulation.

In the other series of experiments described in the present study, we investigated the EPR effect after activation of metastatic tumor cells in the lungs using liposomes encapsulating ICG (which is fluorescent). No ICG fluorescence was detected in the lungs 24 h after the systemic administration of ICG-LP (diameter: 145 ± 7 nm), whereas fluorescence was detected in the solid tumor (Fig. 3B,D). This finding suggests that ICG-LP was cleared from the metastatic LN and lung much more rapidly than from solid tumor. However, ICG accumulation on day 29 increased for FM3A-Luc cells whereas no increase was observed in the KM-Luc/GFP cell groups, which indicated that the EPR effect in early lung metastasis may depend on the primary tumor cell lines. Moreover, histopathology confirmed that tumor neovascularization near metastatic foci was not observed during the early stage of lung metastasis. Thus, the results presented in this study indicated that hematogenous anticancer chemotherapy utilizing the EPR effect might not be useful for the treatment of early-stage lung metastasis.

In conclusion, the distribution of a systemically administered anticancer agent, including small and macromolecules, might be disrupted by the formation of tumor emboli in arterioles after the activation of lung metastasis. Therefore, conventional systemic chemotherapy might be ineffective for the treatment of lung metastasis. In order to improve the prognosis and survival of patients with cancer, it will be necessary to develop a novel therapeutic method for the treatment of pulmonary metastasis that is not affected by metastatic foci in lung arterioles.

## Materials and Methods

### Ethics

All animal experiments were performed in accordance with the institutional guidelines and approved by the Institutional Animal Care and Use Committee of Tohoku University (Permit Number: 2019BeA009 and 2018BeA004).

### Mice

A total of 167 male or female MXH10/Mo/lpr mice (age range: 12–16 weeks of age) were randomized into groups^13,14^. Mice were bred under specific pathogen-free conditions in the Animal Research Institute, Graduate School of Medicine, Tohoku University.

### Cell preparation and inoculation

Luciferase-expressing malignant fibrous histiocytoma-like KM-Luc/GFP cells^20^ and mammary carcinoma FM3A-Luc cells^14^ were used. On the inoculation day (defined as day 0), cells were shown to test negative for *Mycoplasma* using a commercial kit (Lonza Rockland, Inc., Rockland, ME, US). All cell implantation procedures were performed under general anesthesia (inhalation of 2.5% isoflurane in oxygen; Abbott, Wiesbaden, Germany) by a surgical method previously described^16–19^. On day 0, KM-Luc/GFP cells (*n* = 51) and FM3A-Luc cells (*n* = 72) were suspended at a concentration of 3.3 × 10^5^ cells/mL in a mixture of 20 μL phosphate-buffered saline (PBS) and 40 μL of 400 mg/mL Matrigel (Collaborative Biomedical Products, Bedford, MA, USA). Cells were inoculated into the SiLN manually.

### Lung metastasis induction and detection

The experimental procedures were conducted as previously described^16–19^. Mice were placed in a supine position on a heated stage and anesthetized. After depilation and skin disinfection, a minimally invasive approach was used for incision of the skin and exposure and extirpation of the tumor-bearing SiLN on day 3 post-inoculation for both cell types. Metastasis to the PALN and lung were assessed using the IVIS^18^ at 6 h and on days 3, 6 and 9 post-inoculation of KM-Luc/GFP cells and days 7, 14, 21 and 28 post-inoculation of FM3A-Luc cells. Luciferin (15 mg/kg; Promega, Madison, WI, USA) was injected intraperitoneally into each mouse. At 10 min after the luciferin injection, bioluminescence images were captured for 30 sec (KM-Luc/GFP group) or 60 sec (FM3A-Luc group). Induction of metastasis was considered to have occurred if the luciferase intensity was more than the background level (4 × 10^4^ p/s/cm^2^/sr for the KM-Luc/GFP group and 5 × 10^5^ p/s/cm^2^/sr for the FM3A-Luc group). Although metastasis in the lung was not detected by *in vivo* bioluminescence imaging in some mice, it was subsequently confirmed by *ex vivo* bioluminescence imaging and/or histology in these mice. The incidence of metastasis in the lung was 67% for FM3A-Luc cells and 61% for KM-Luc/GFP cells. Only mice with confirmed lung metastasis were used in experiments evaluating the EPR effect and pulmonary vascularization changes.

### Micro-CT scanning and post-processing

The experimental procedures were conducted as previously described^21,22^. The lungs were placed in a high-resolution micro-CT scanner (Scanmate-X090, Comscantecno Co., Kanagawa, Japan) and scanned. The micro-CT-generated DICOM files were analyzed using Amira software (versions 5.6 or 6.0; FEI, Hillsboro, OR, US). To examine the morphometry of the perfused lung vessels, a three-dimensional (3D) skeleton was generated from the 2D micro-CT data for the segmented tissue using the methods described by Lee *et al*.^23^. Threshold segmentation identified the material of interest, and this was optimized according to the specimen^24^.

### ICG-LP leakage from the vasculature in a solid tumor and lung

ICG-LP was prepared as described in the supplementary Information: the average diameter was 145.0 ± 6.8 nm, and the average zeta potential was −5 ± 2 mV (Fig. S1C,D). FM3A-Luc cells (1.0 × 10^7^ cells/mL) in 100 μL PBS (Collaborative Biomedical Products) was subcutaneously injected into the rear flank (*n* = 12) to make a solid tumor. A volume of 200 μL of ICG or ICG-LP (lipid concentration: 1 mg/mL) was injected into the tail vein on day 11 post-inoculation (*n* = 4, per group). The fluorescence intensity of leaked ICG-LP was measured at 0 min, 5 min, 1 h, 6 h, 24 h and 48 h using the IVIS.

### ICG-LP distribution in normal mice with and without SiLN resection

ICG-LP was intravenously injected into control mice (*n* = 4 per group), and its biodistribution was evaluated after non-resection or resection of the SiLN. Mice were humanely euthanized 24 h after ICG-LP injection, and the accumulation of ICG-LP in the excised organs was measured *ex vivo* using the IVIS.

### Histological analysis

The excised organs were harvested on days 6, 9 and 18 for KM-Luc/GFP cells (*n* = 25) and on days 7, 14, 21 and 28 for FM3A-Luc cells (*n* = 34). The tissue samples were fixed, dehydrated and embedded in paraffin. The excised organs from mice in the control group (*n* = 4) were obtained on day 6 post-inoculation. Embedded specimens were serially sectioned (2 µm) and stained with EM or HE or immunostained for CD31 or Ki67^18^. Anti-CD31 staining to identify vascular endothelial cells was performed using an automated processor (Discovery, Ventana Medical Systems, Inc., Tucson, AZ, USA). The areas that contained or did not contain tumor were classified manually based on the histological findings.

### Statistical analysis

Statistical significance was determined using one-way analyses of variance (ANOVA) and the Kruskal-Wallis test, unless otherwise stated. A *P*-value ≤ 0.05 was considered to be statistically significant. All measurements are presented as the mean ± standard error of the mean (SEM). Statistical analyses were carried out using Excel 2016 (Microsoft Corp., Redmond, WA, US) and Prism 7 (GraphPad Software, Inc., La Jolla, CA, US).

## Supplementary information

Supplementary materials and methods

Video S1

Video S2

Video S3

Video S4

Video S5

Video S6

## Data Availability

The data that support the findings of this study are available from the corresponding author upon reasonable request.
