# Supplementary material for: Analysis of tumor vascularization in a mouse model of metastatic lung cancer
Source: Sci Rep. 2019 Nov 5;9:16029. doi: 10.1038/s41598-019-52144-2 (PMC6831815; doi:10.1038/s41598-019-52144-2)
Supplement: Supplementary file 1 — Supplementary materials and methods [file 41598_2019_52144_MOESM1_ESM.pdf]

Analysis of tumor vascularization in a mouse model of metastatic lung cancer

(Supplementary information)

Ariunbuyan Sukhbaatar[1,2], Maya Sakamoto[3], Shiro Mori[1,2,4], Tetsuya Kodama[1,2]

[1]Laboratory of Biomedical Engineering for Cancer, Graduate School of Biomedical Engineering, Tohoku University, 4-1 Seiryo, Aoba, Sendai, Miyagi 980-8575, Japan

[2]Biomedical Engineering Cancer Research Center, Graduate School of Biomedical Engineering, Tohoku University, 4-1 Seiryo, Aoba, Sendai, Miyagi 980-8575, Japan

[3]Department of Oral Diagnosis, Tohoku University Hospital, 1-1 Seiryo, Aoba, Sendai, Miyagi 980-8574, Japan

[4]Department of Oral and Maxillofacial Surgery, Tohoku University Hospital, 1-1 Seiryo, Aoba, Sendai, Miyagi 980-8574, Japan

Supplemental Methods

All animal experiments were performed in accordance with the institutional guidelines and approved by the Institutional Animal Care and Use Committee of Tohoku University (Permit Number: 2019BeA009 and 2018BeA004).

Ex vivo analysis of luciferase activity

Experimental mice were humanely euthanized under anesthesia (inhalation of 2.5% isoflurane in oxygen) on day 6 for the control group, days 6, 9 and 18 for the KM-Luc/GFP group and days 7, 14, 21 and 28 for the FM3A-Luc group, and the lungs and PALN were harvested. *Ex vivo* bioluminescence imaging of the excised organs was performed immediately after the *in vivo* analyses for each mouse.

Contrast agent preparation and characterization

The gelatin-based barium contrast agent was prepared as follows: 50.0 g barium sulfate (Enemaster Enema, Fushimi Pharmaceutical Co., Kagawa, Japan) was mixed with 3.1 g of gelatin (Cook gelatin, Morinaga & Co., Tokyo, Japan), 5.7 mL of orange acrylics (Liquitex; cadmium red medium, soft type, bonnyColArt Co., Tokyo, Japan), 5.7 mL of heparin (1,000 units/mL, Mochida Pharmaceutical Co., Tokyo, Japan) and dissolved in 78.0 mL of warmed saline (50°C). The mixture was pre-heated for 1 h before perfusion at 37°C. The contrast size was 602 ± 144 nm and its zeta potential was -48 ± 1 mV at 37°C (measured using an ELSZ-2 particle size and zeta potential analyzer, Otsuka Electronics, Osaka, Japan) (Fig. S1A and S1B).

Perfusion and specimen preparation

MXH10/Mo/lpr mice (12–16 weeks of age) were used to investigate vascularization changes in the lungs of control mice (non-resection and resection of a SiLN), a KM-Luc/GFP group (non-resection and resection of a tumor-bearing SiLN) and an FM3A-Luc group (non-resection and resection of a tumor-bearing SiLN) (n = 6 per group). Control mice in which the SiLN was either resected or non-resected were used to explore whether the surgical procedures influenced pulmonary vascularization. The groups inoculated with KM-Luc/GFP or FM3A-Luc cells into the SiLN were each divided into two subgroups, namely a SiLN non-resection group and a SiLN resection group. After anesthesia with 2% isoflurane, mice were individually heparinized (Mochida Co. Ltd) by intravenous injection (100 µL/mouse, 1,000 U/mL), and papaverine hydrochloride (Nichi-Iko Co. Ltd, Toyama, Japan) was injected subcutaneously (50 µL/mouse, 40 mg/mL) into the scruff of the neck according to institutional protocols. Postmortem intracardiac perfusion was achieved with the following surgical procedures: a transverse skin incision across the lower half of the chest was made using small scissors, and the diaphragm was severed peripherally to expose the thoracic cavity [1]. The heart was cannulated distally at the apex of the left ventricle with a 27-G butterfly needle, and the inferior vena cava between the diaphragm and liver was severed to provide an outflow. To facilitate blood clearance, 4 mL of PBS and 4 mL of 10% formalin were administered with a syringe pump set at a rate of delivery of 300 µL/min. As a result, peripheral organs became visibly blanched and hardened. The perfusion medium was then changed to the gelatin-based barium contrast agent at a viscosity appropriate for small vessel filling. At the conclusion of the procedure, the mice were placed in a refrigerator to ‘cure’ for 1 h. Harvested lungs were fixed in 10% formalin for 4 days at 4°C and stained with

hematoxylin-eosin (HE).

Preparation of indocyanine green liposomes

Indocyanine green liposomes (ICG-LP) were used for evaluation of the enhanced permeability and retention (EPR) effect in solid tumors and metastatic PALNs as previously described [2]. The threshold of the EPR effect was set at a diameter of 200 nm [3]. The diameter and zeta potential of the ICG-LP were measured using a particle size and zeta potential analyzer (ELSZ-2, Otsuka Electronics, Osaka, Japan). The average values of the size and zeta potential were calculated using 8 independent measurements for each sample: the average diameter was 145.0 ± 6.8 nm, and the average zeta potential was -5 ± 2 mV (Fig. S1C and S1D).

Leakage of ICG-LP in the PALN and lung

ICG-LP (200 µL) was injected intravenously into the tail on day 6 for the control group, on days 6, 9 and 18 post-inoculation of KM-Luc/GFP cells (*n* = 12) and on days 7, 14, 21 and 28 post-inoculation of FM3A-Luc cells (*n* = 31). The IVIS was used to measure the fluorescence intensity of any leaked ICG-LP at 5 min, 30 min, 2 h, 6 h and 24 h post-injection of ICG-LP; a pre-injection measurement was also made.

Ex vivo analysis of ICG-LP accumulation

Mice were humanely euthanized 24 h after the ICG-LP injection (on day 7 for the control group, on days 7, 10 and 19 for the KM-Luc/GFP group, and on days 8, 15, 22 and 29 for the FM3A-Luc group), and the lungs and PALN were then harvested. Measurements were taken and samples frozen in liquid nitrogen. The frozen organs were homogenized in 1

mL of PBS using a T25 basic Ultra Turrax S1 (IKA® Works Inc., Wilmington, Ohio, US). Next, 500 µL of the supernatant was transferred into a 48-well plate, the fluorescence intensity was measured using the IVIS, and the fluorescence intensity multiplied by 2 for calculation of ICG accumulation (since only half of the 1 mL sample was assayed).

Evaluation of ICG-LP retention in the metastatic lymph node and metastatic lung

Four MXH10/Mo/lpr mice (aged 12–16 weeks) were used to evaluate the retention of ICG-LP. Mice were killed at different time periods after the intravenous injection of ICG-LP, and the retention of ICG-LP in the metastatic lymph node and metastatic lung (n = 2 per group) was evaluated. Mice were humanly euthanized 0 min, 10 min or 30 min after the injection of ICG-LP, and ICG-LP retention in the excised organs was measured *ex vivo* using the IVIS.

Adverse effects

Water and food consumption, behavior, respiration and stool tests were observed daily. Anesthetic and post-operative effects were checked the next morning after resection of the tumor-bearing SiLN. Body weight was measured on each day of the experimental procedure. Significant weight loss was considered to be > 10% of body weight.

Figure S1. Characterization of gelatin-based barium contrast agent and indocyanine green liposomes (ICG-LP).

(A) Particle size and (B) zeta potential of the gelatin-based barium contrast agent. The average diameter of the gelatin-based barium contrast agent was 602 ± 144 nm (n = 2), and the zeta potential was -48 ± 1 mV (n = 2).

(C) Particle size and (D) zeta potential of ICG-LP. The average diameter of ICG-LP was 145.0 ± 6.8 nm (*n* = 12), and the zeta potential was -5 ± 2 mV (*n* = 9).

Figure S2. Evaluation of the enhanced permeability and retention (EPR) effect in solid tumor and biodistribution of indocyanine green liposomes (ICG-LP) in non-tumor bearing mice with and without resection of the subiliac lymph node (SiLN).

A, The EPR effects in solid tumor (mouse mammary carcinoma FM3A-Luc cells) were shown after injections of either indocyanine green (ICG) (Aa, Ab) or ICG-liposome (ICG-LP) (Ac, Ad) (*n* = 4 per group). Aa, Ac: Luciferase activity of the solid tumor in a mouse. Ab, Ad: Changes in fluorescence intensity with time.

The maximal fluorescence intensity was detected 5 min after the injection of ICG or ICG-LP (Ab, Ad). This result suggested that free ICG and ICG-LP spread throughout the whole body after injection.

B, C. ICG-LP was injected into control mice in which the SiLN had either not been resected (Ba, Bb) or resected (Ca, Cb) (*n* = 4 per group). Bioluminescence images of control mice (non-resection and resection groups) are shown before ICG-LP injection (Ba, Cc). Fluorescence intensity was measured at different times. No fluorescence was detected in either group 24 h after ICG-LP injection (Bb, Cb).

D. Organs in B, C were removed 24 h post-injection of ICG-LP and their fluorescence intensity was measured. The fluorescence intensity of the lungs did not differ between the non-resection and resection groups. The fluorescence intensities of the spleen and proper axillary lymph node (PALN) were significantly higher in the non-resection group than in the resection group (Kruskal-Wallis test: ****$P < 0.0001$). Data are presented as the mean ± SEM.

Figure S3. Quantitative *ex vivo* measurement of luciferase activity and indocyanine group (ICG) accumulation in the proper axillary lymph node (PALN).

PALNs were harvested 24 h after ICG-LP injection, and ICG accumulation was quantified from the *ex vivo* PALN fluorescence intensity as ng of ICG per g of tissue.

A. Luciferase activity in the PALN measured *ex vivo* on days 6, 9 and 18 post-inoculation of KM-Luc/GFP cells (day 6, n = 5; day 9, n = 4; day 18, n = 6) and on day 6 for the control group (n = 4). The highest luciferase activity in the PALN was detected on day 18. Kruskal-Wallis test: *$P < 0.05$, control *vs* day 6 and control *vs* day 18. Data are presented as the mean ± SEM.

B. ICG accumulation in the PALN in the KM-Luc/GFP group. PALNs were harvested 24 h after ICG-LP injection, i.e., on days 7, 10 and 19 for the KM-Luc/GFP group (day 6, n = 8; day 9, n = 7; day 18, n = 9) and on day 7 for the control group (n = 4). No significant differences were observed between groups. Data are presented as the mean ± SEM.

C. Luciferase activity in the PALN measured *ex vivo* on days 7, 14, 21 and 28 post-inoculation of FM3A-Luc cells (day 7, n = 11; day 14, n = 10; day 21, n = 10; and day 28, n = 9) and on day 6 for the control group (n = 4). Numerically, the highest luciferase activity in the PALN was detected on day 28, although there were no significant differences between groups. Data are presented as the mean ± SEM.

D. ICG accumulation in the PALN of the FM3A-Luc group. PALNs were harvested 24 h after ICG-LP injection, i.e., on days 8, 15, 22 and 29 for the FM3A-Luc group (day 7, n = 6; day 14, n = 5; day 21, n = 5; day 28, n = 5) and on day 7 for the control group (n = 4). No significant differences were observed between groups. Data are presented as the

mean ± SEM.

Figure S4. Evaluation of metastasis to the PALN.

A–D. Experiments utilizing KM-Luc/GFP cells.

E–I. Experiments utilizing FM3A-Luc cells.

Tumor cells were inoculated into the unilateral SiLN, and the SiLN was resected on day 3 post-inoculation to activate tumor cells in the lungs. ICG-LP was intravenously injected into the tail vein on different experimental days, and the PALN was harvested 24 h after ICG-LP injection to evaluate the distribution of ICG-LP.

Representative *ex vivo* bioluminescence images of the PALN are shown in A–D (KM-Luc/GFP cells) and E–I (FM3A-Luc cells). Metastasis in the PALN was confirmed on days 9 and 18 for the KM-Luc/GFP group and on days 7, 21 and 28 for the FM3A-Luc group. Representative *ex vivo* images of ICG-LP biofluorescence are presented in a1–d1 (KM-Luc/GFP cells) and e1–i1 (FM3A-Luc cells). No ICG-LP fluorescence was detected in the PALN for either tumor cell type.

a2–i2: hematoxylin-eosin (HE) staining of the PALN. a3–i3: immunostaining of CD31 in the PALN. a2–d2 and a3–d3: KM-Luc/GFP cells. e2–i2 and e3–i3: FM3A-Luc cells. Scale bar: 20 µm; T, tumor.

No tumor cells were found in the PALN of the control group for either tumor cell type. Metastasis was detected in the PALN on day 9 (c2) and day 18 (d2) for the KM-Luc/GFP group and on day 7 (f2), day 21 (h2) and day 28 (i2) for the FM3A-Luc group.

Figure S5. Normalized body weight changes with time.

A. Subiliac lymph node (SiLN) inoculated with KM-Luc/GFP cells.

Control (n = 4), day 6 (n = 12), day 9 (n = 13) and day 18 (n = 8).

There were no significant differences between groups and no severe weight loss occurred during the experimental period. Data are given as the mean ± SEM.

B. SiLN inoculated with FM3A-Luc cells.

Control (n = 4), day 7 (n = 11), day 14 (n = 10), day 21 (n = 13), and day 28 (n = 14).

There were no significant differences between groups, and no severe weight loss occurred during the experimental period. Data are presented as the mean ± SEM.

Video S1. Reconstructed 3D cast movies of the pulmonary vasculature. The SiLN was not inoculated with tumor cells and not resected.

Video S2. Reconstructed 3D cast movies of the pulmonary vasculature. The SiLN was not inoculated with tumor cells and was resected.

Video S3. Reconstructed 3D cast movies of the pulmonary vasculature. The SiLN was inoculated with FM3A-Luc cells and not resected.

Video S4. Reconstructed 3D cast movies of the pulmonary vasculature. The SiLN was inoculated with FM3A-Luc cells and resected.

Video S5. Reconstructed 3D cast movies of the pulmonary vasculature. The SiLN was inoculated with KM-Luc/GFP cells and not resected.

Video S6. Reconstructed 3D cast movies of the pulmonary vasculature. The SiLN was inoculated with KM-Luc/GFP cells and resected.

Figure S1

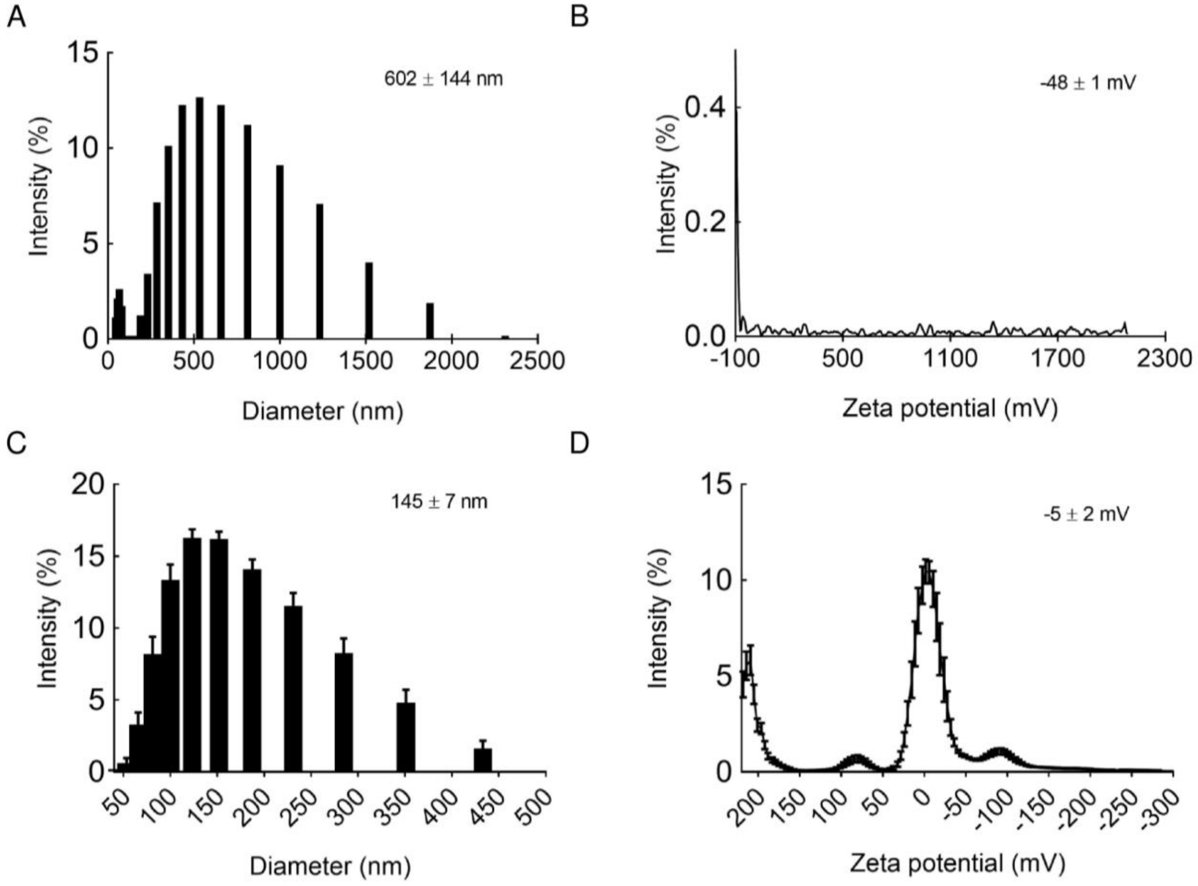
A
B
C
D
602 ± 144 nm
-48 ± 1 mV
145 ± 7 nm
-5 ± 2 mV
Intensity (%)
Diameter (nm)
Zeta potential (mV)

Figure S2

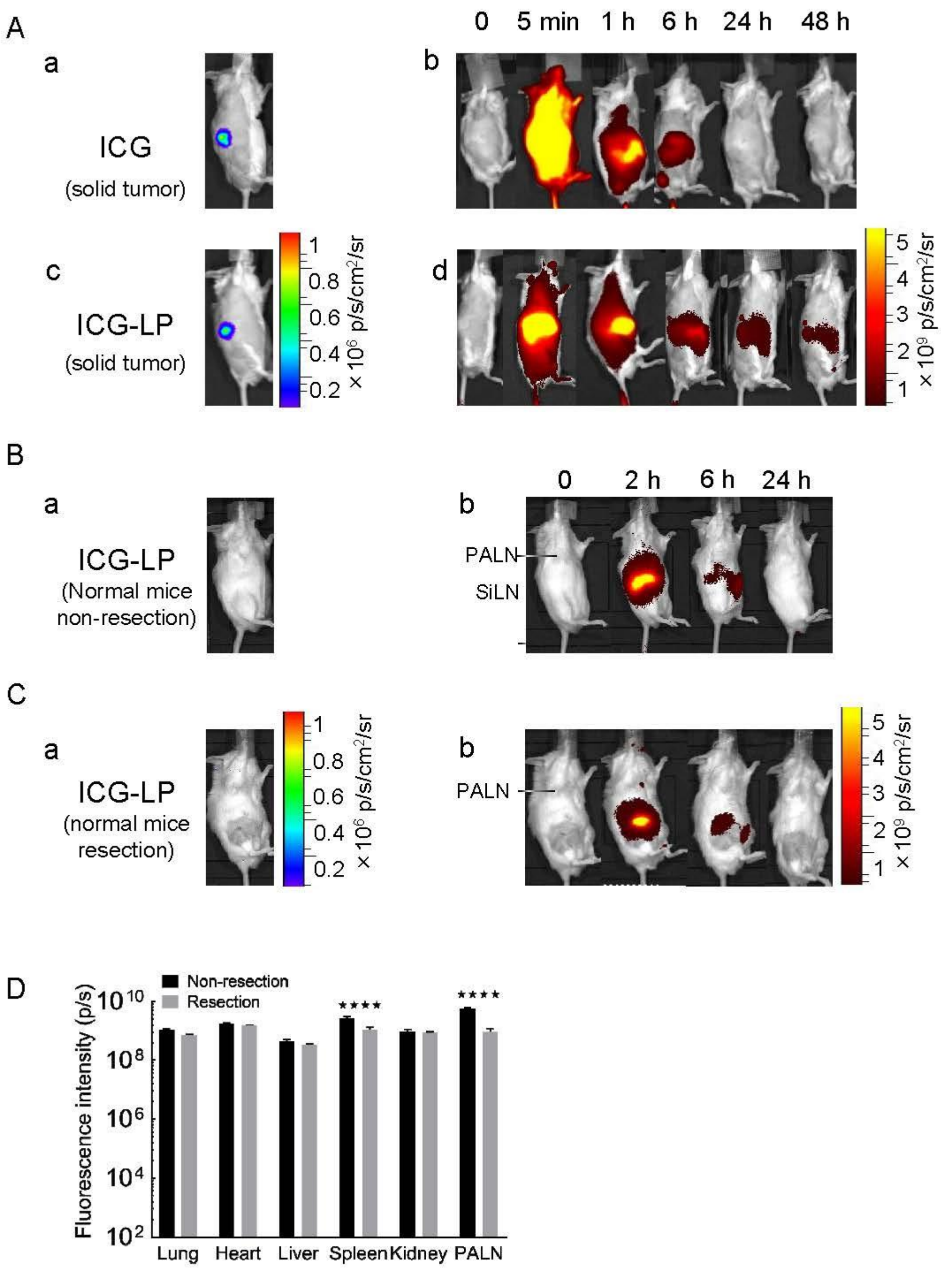
A
a
ICG
(solid tumor)
0
5 min
1 h
6 h
24 h
48 h
b
c
ICG-LP
(solid tumor)
1
0.8
0.6
0.4
0.2
× 10⁶ p/s/cm²/sr
d
5
4
3
2
1
× 10⁹ p/s/cm²/sr
B
a
ICG-LP
(Normal mice
non-resection)
b
0
2 h
6 h
24 h
PALN
SiLN
C
a
ICG-LP
(normal mice
resection)
b
PALN
D
Non-resection
Resection
Fluorescence intensity (p/s)
10¹⁰
10⁸
10⁶
10⁴
10²
****
****
Lung
Heart
Liver
Spleen
Kidney
PALN

Figure S3

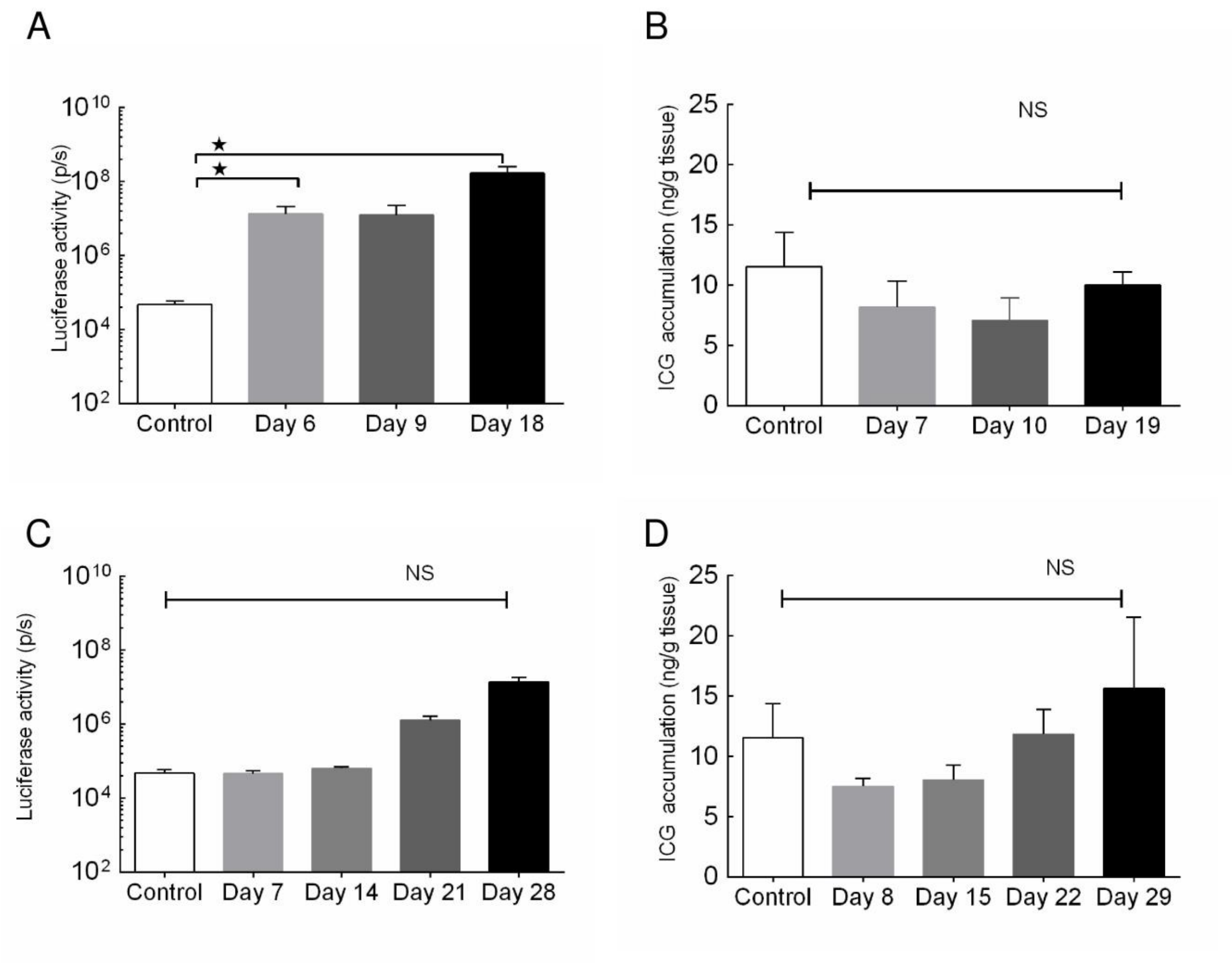
A
10^{10}
10^8
10^6
10^4
10^2
Luciferase activity (p/s)
★
★
Control
Day 6
Day 9
Day 18
B
25
20
15
10
5
0
ICG accumulation (ng/g tissue)
NS
Control
Day 7
Day 10
Day 19
C
10^{10}
10^8
10^6
10^4
10^2
Luciferase activity (p/s)
NS
Control
Day 7
Day 14
Day 21
Day 28
D
25
20
15
10
5
0
ICG accumulation (ng/g tissue)
NS
Control
Day 8
Day 15
Day 22
Day 29

Figure S4

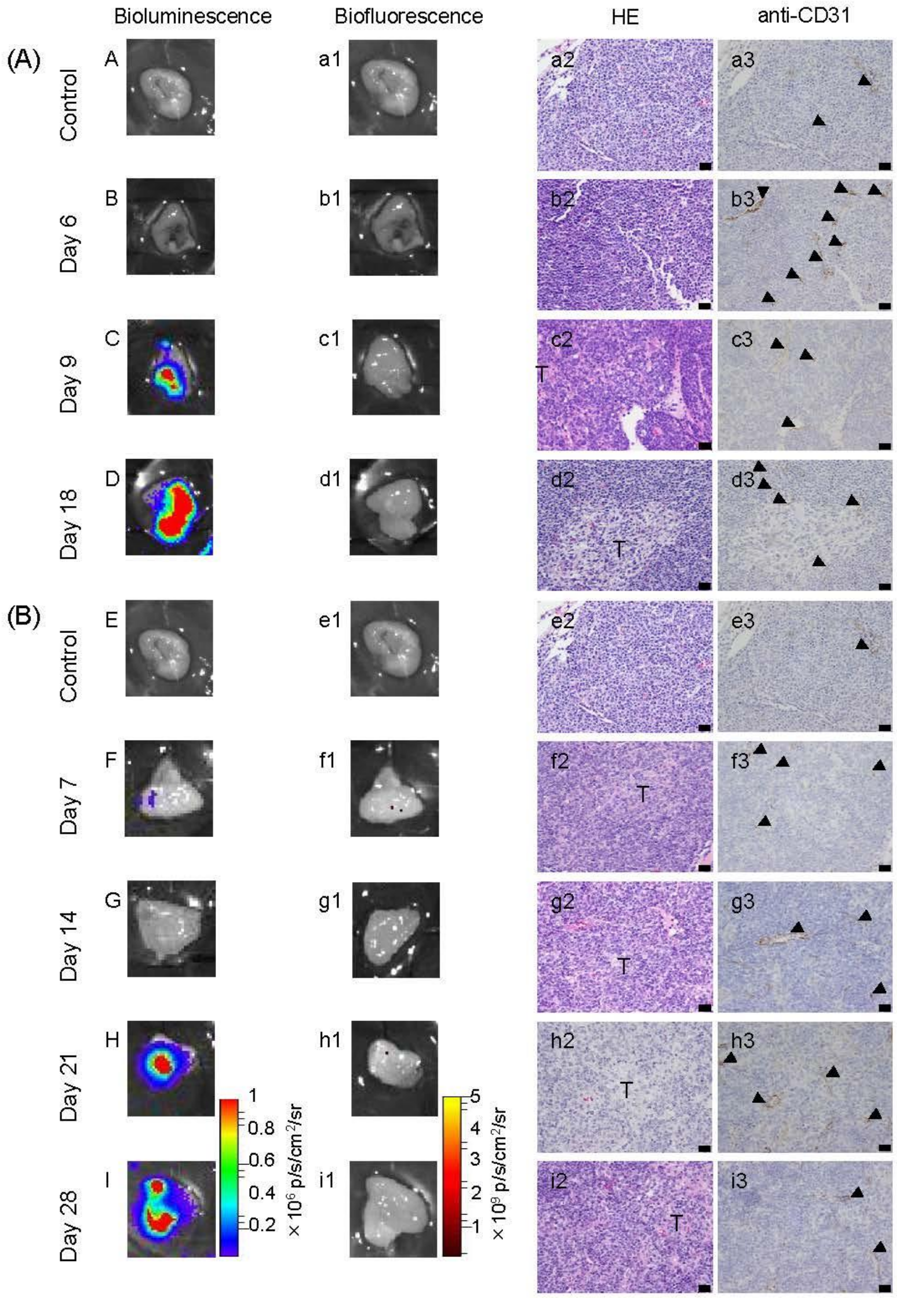
Bioluminescence
Biofluorescence
HE
anti-CD31
(A)
Control
Day 6
Day 9
Day 18
(B)
Control
Day 7
Day 14
Day 21
Day 28
A
a1
a2
a3
B
b1
b2
b3
C
c1
c2
c3
D
d1
d2
d3
E
e1
e2
e3
F
f1
f2
f3
G
g1
g2
g3
H
h1
h2
h3
I
i1
i2
i3
T
1
0.8
0.6
0.4
0.2
× 10^6 p/s/cm^2/sr
5
4
3
2
1
× 10^9 p/s/cm^2/sr

Figure S5

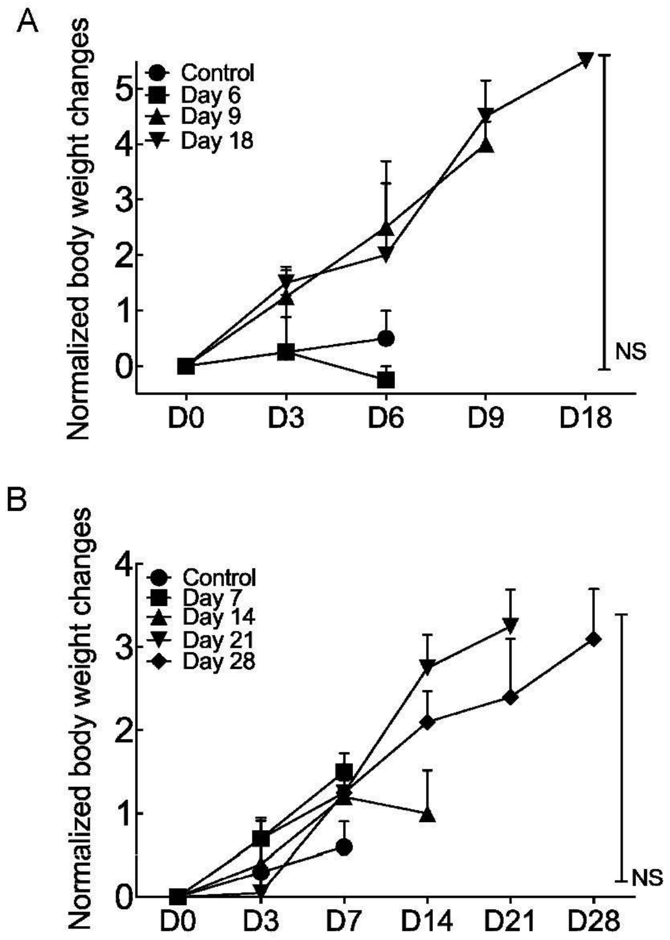
A
Normalized body weight changes
Control
Day 6
Day 9
Day 18
NS
D0
D3
D6
D9
D18
B
Normalized body weight changes
Control
Day 7
Day 14
Day 21
Day 28
NS
D0
D3
D7
D14
D21
D28

References

1 Vasquez, S. X. *et al.* Optimization of microCT imaging and blood vessel diameter quantitation of preclinical specimen vasculature with radiopaque polymer injection medium. *PLoS One* **6**, e19099 (2011).

2 Mikada, M. *et al.* Evaluation of the enhanced permeability and retention effect in the early stages of lymph node metastasis. *Cancer. Sci.* **108**, 846-852 (2017).

3 Liu, D., Mori, A. & Huang, L. Role of liposome size and RES blockade in controlling biodistribution and tumor uptake of GM1-containing liposomes. *Biochim. Biophys. Acta* **1104**, 95-101 (1992).
